# Single-nucleus analysis of thoracic perivascular adipose tissue reveals critical changes in cell composition, communication, and gene regulatory networks induced by a high fat hypertensive diet

**DOI:** 10.1101/2025.02.13.636878

**Published:** 2025-02-14

**Authors:** Leah Terrian, Janice M. Thompson, Derek E. Bowman, Vishal Panda, G. Andres Contreras, Cheryl Rockwell, Lisa Sather, Gregory D. Fink, D. Adam Lauver, Rance Nault, Stephanie W. Watts, Sudin Bhattacharya

**Affiliations:** 1. Department of Biomedical Engineering, Michigan State University, East Lansing, MI, USA; 2. Department of Pharmacology and Toxicology, Michigan State University, East Lansing, MI, USA; 3. Institute for Quantitative Health Science and Engineering, Michigan State University, East Lansing, MI, USA; 4. College of Osteopathic Medicine, Michigan State University, East Lansing, MI, USA; 5. Department of Large Animal Clinical Sciences, Michigan State University, East Lansing, MI, USA; 6. Institute for Integrative Toxicology, Michigan State University, East Lansing, MI, USA; 7. Denotes individuals contributed equally as first authors to this work; 8. Denotes lead investigators/funding

## Abstract

Cardiovascular disease (CVD) is the leading cause of death worldwide, with hypertension being its primary causal factor. Most blood vessels are surrounded by perivascular adipose tissue (PVAT), which regulates blood vessel tone through the secretion of vasoactive factors. PVAT is recognized as a key mediator of vascular function and dysfunction in CVD, although the underlying mechanisms remain poorly understood. To investigate PVAT’s mechanistic role in hypertension, we performed single nucleus RNA-Sequencing analysis of thoracic aortic PVAT from Dahl SS rats fed a high-fat, hypertensive diet. Computational analysis revealed extensive diet-induced changes in cell-type composition, cell-type specific gene expression, cell-cell communication pathways, and intracellular gene regulatory networks within PVAT. Furthermore, we identified key transcription factors mediating these networks and demonstrated through virtual knock-out experiments that these factors could serve as potential therapeutic targets for preventing or reversing PVAT’s hypertensive state.

## INTRODUCTION

Cardiovascular diseases (CVDs) were the cause of more than 20.5 million deaths globally in 2021 alone^1^. Hypertension is the most prevalent CVD with 1.1 billion people affected and is considered the entree to other CVDs such as heart failure, coronary artery disease, stroke, and peripheral artery disease^2^. Hypertension is especially problematic in obese and overweight subjects where the development of high blood pressure has been directly linked to the expansion of perivascular adipose tissue (PVAT)^3^. The PVAT or tunica adiposa is the outermost layer of tissue surrounding most blood vessels. Long considered to be a passive tissue involved in lipid storage and mechanical support, PVAT is now recognized as an active contributor to vascular function, playing important roles both in physiological homeostasis and its perturbation in metabolic and cardiovascular disease^4–7^. Adipocytes, the predominant cell type in PVAT, secrete various molecules including adipokines and cytokines, thereby modulating the functioning of smooth muscle and endothelial cells through paracrine signaling^5^. Additionally, they play a key role in regulation of aortic stiffness, as demonstrated by *ex vivo* mechanical studies^8^.

PVAT remains an enigmatic tissue whose precise mechanistic role in vascular dysfunction is not well understood. In healthy tissue, PVAT exerts an anticontractile effect on blood vessels, thereby helping maintain vascular tone and blood pressure homeostasis. However, this benefit of PVAT is lost in many cardiovascular diseases, including hypertension^4^, where changes in the physiological function of PVAT have been observed in arteries from both rodents and humans^9–11^. We focus here on possible alterations in PVAT phenotype in response to a high fat (HF) diet that causes an elevation in blood pressure in a rat model. It is currently unknown whether the constituent cell types in PVAT undergo changes in gene expression in response to hypertension in the context of excessive adiposity. We aim to address this gap by exploring several questions using our data. Do the diverse cell types in PVAT change uniformly when challenged with HF diet, or are the changes cell type-specific? How do crucial patterns of cell-cell communication adapt in response to an HF diet? What gene regulatory networks mediate these changes? Are the observed changes sex-specific, and importantly, are they reversible?

We examined these questions with novel single-nucleus RNA sequencing (snRNA-seq) of thoracic aortic PVAT (taPVAT) from Dahl salt sensitive (Dahl SS) rats fed a HF diet. This rat model, developed by us and others^12^, reproducibly develops elevated blood pressure and increased visceral adiposity when fed a HF diet (60% kcal fat) from weaning, compared to age-matched rats fed a control diet (10% kcal fat). Moreover, the HF diet causes hypertension in both male and female Dahl SS rats. This is thus a good model for hypertension in humans, where up to 70% of those with hypertension are obese, defined as a Body Mass Index of 30 or above^13^.

We characterized time- and sex-specific alterations caused by HF diet in the proportions of different constituent cell types in PVAT: adipocytes, endothelial cells, fibroblasts, mesothelial cells, immune cells, pericytes, smooth muscle cells, and neuronal cells. Additionally, individual cell types showed characteristic changes in gene expression patterns induced by diet, in a manner dependent on both sex and duration on diet. Analyzing cell-cell communication within PVAT, we found that while there were broad similarities in inferred signaling pathways across sex and diet at the level of cell types in aggregate, there was considerable heterogeneity in signaling mechanisms both within and between cell types at the level of individual cells. Finally, gene regulatory network analysis with CellOracle^14^ identified key transcriptional drivers of changes in adipocyte gene expression and revealed potential molecular targets to suppress or possibly reverse diet-dependent alterations in gene expression.

## RESULTS

### Both male and female Dahl SS rats exhibit increased blood pressure when fed a high fat diet

**Extended Data Figure 1:**
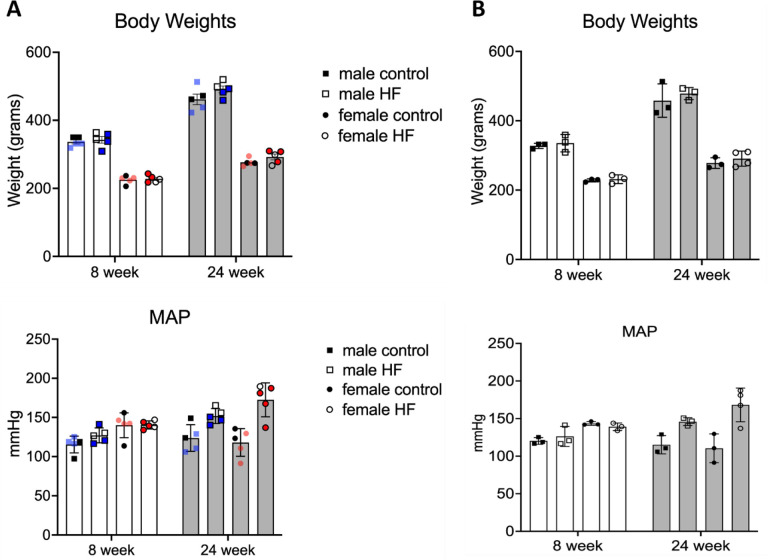
**(A)** Body weights of male and female Dahl SS rats on control and high fat diet in cohorts at 8 (N=5) and 24 weeks (N=5) (left); subset of body weights from rats on control and high fat diet used in reported snRNA-seq experiments at 8 (N=3) and 24 weeks (N=3–4) (right). (**B)** MAP of male and female Dahl SS rats on control and high fat diet in cohorts at 8 (N=5) and 24 weeks (N=5) (left); subset of MAP from rats on control and high fat diet used in reported snRNAseq experiments at 8 (N=3) and 24 weeks (N=3–4) (right). Colored symbols indicate samples (N=3–4) used for snRNAseq work. Bars represent means ± SEM.

We first evaluated the difference in body weight and mean arterial pressure (MAP) between male and female Dahl SS rats at 8 and 24 weeks on control and HF diet. Male and female rats at 8 weeks on diet showed no significant change in body weights between the control and HF diet fed groups (Extended Data Figure 1). At 24 weeks, there was a modest increase in weight in both HF male and female animals compared to the control diet fed groups. Compared to their respective 8-week groups, MAPs were found to be lower in the control diet fed rats but higher in the HF diet fed rats at 24 weeks in both males and females. This elevation in blood pressure in the HF diet fed Dahl SS rats was an expected result as previously documented^12^.

### Thoracic aortic PVAT is composed of distinct cell subtypes

**Figure 1:**
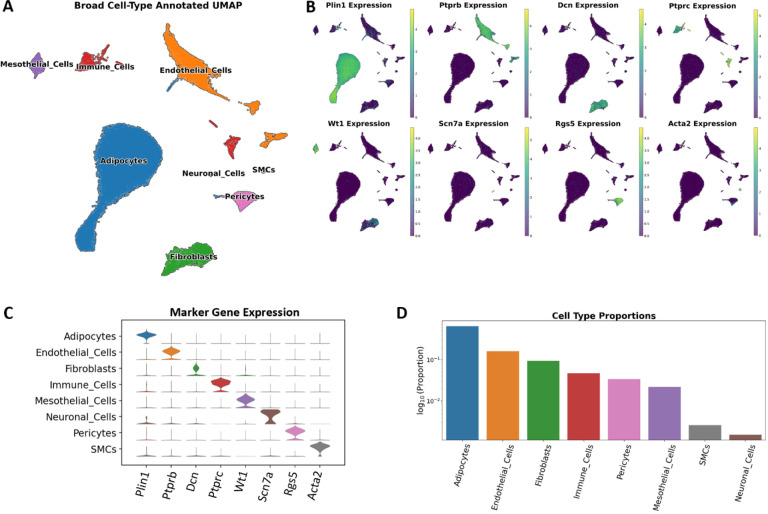
High level overview of cell types found in the whole dataset. **A.** Broad, high-level, cell type annotated UMAP plot of all 71,813 cells inclusive of all samples. **B.** Locations of marker gene expression on the UMAP. **C.** Stacked violin plot of marker gene expression by high-level cell type annotation. **D.** The proportion of each high-level cell type inclusive of all samples. Note that the y-axis is in log-scale.

Given that taPVAT is a complex tissue made up of varied cell types, we wanted to thoroughly examine cell type composition in our data. A total of twenty-five taPVAT samples from individual Dahl SS rats were used for library preparation and sequencing. Three individual samples were taken of each treatment group except for the 24-week HF diet fed female rat group which had four samples collected due to a sample clog resulting in a smaller number of recovered nuclei. In total, 83,849 nuclei were sequenced with a median of 3159 nuclei per sample. A median of 2501 reads and 1462 genes were detected per nucleus. Ambient RNA accounted for 4.1% of reads and was removed using SoupX^15^. Doublet detection and removal was performed with scDblFinder^16^ which labeled 6.1% of cells as doublets. After quality control and preprocessing, we had a remaining total of 71,813 cells and 20,743 genes for downstream analyses. We subsequently performed Principal Component Analysis (PCA)^17^ and Uniform Manifold Approximation and Projection (UMAP)^18^ to reduce the dimensionality of the data for visualization. The Leiden algorithm^19^ was then implemented at a resolution of 1.6 to cluster data for subsequent cell annotation. The resulting Leiden clusters were labeled by examining the expression of marker genes for cell types that have been previously reported to be found in adipose tissue (Figure 1A – 1B). Initial annotation of clusters was performed at low resolution and identified eight major cell types: adipocytes (*Plin1+*), endothelial cells (ECs, *Ptprb+*), fibroblasts (*Dcn*+), mesothelial cells (*Wt1*+), immune cells (*Ptprc*+), pericytes (*Rgs5*+), smooth muscle cells (SMCs, *Acta2*+), and neuronal cells (*Scn7a*+) (Figure 1C). Not surprisingly, taPVAT is primarily composed of adipocytes (64.1%), endothelial cells (16.0%) and fibroblasts (9.3%), with smaller populations of immune cells (4.7%), pericytes (3.4%), mesothelial cells (2.2%), smooth muscle cells (0.3%), and neuronal cells (0.2%) (Figure 1D). The substantial presence of endothelial cells highlights the richness of the PVAT microvasculature, as detailed in a recent publication by Watts et al^20^.

**Figure 2:**
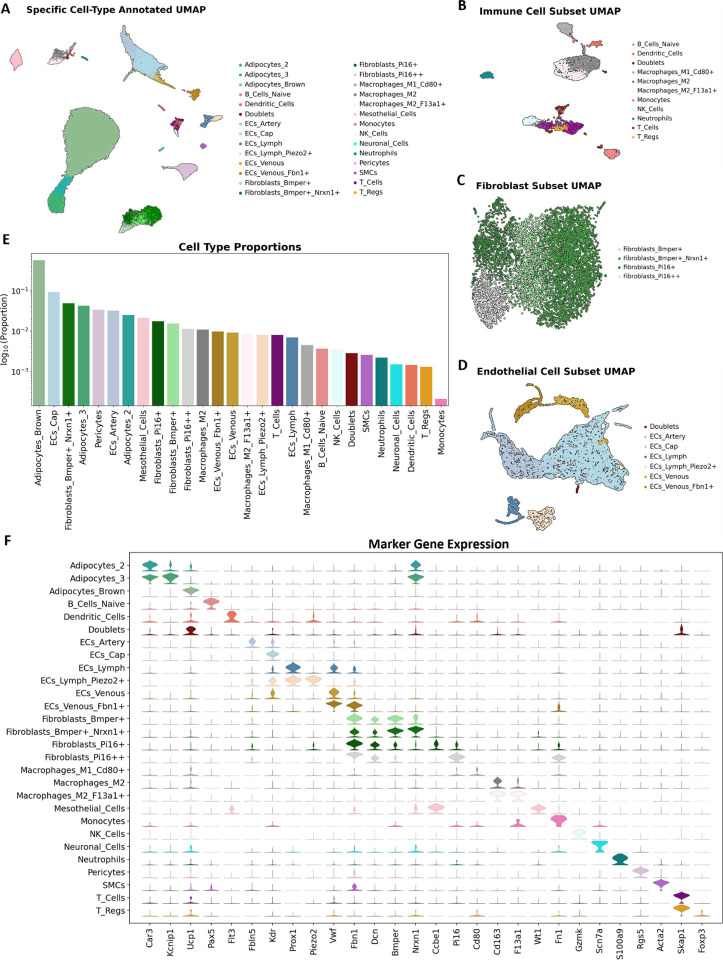
Developing a higher-resolution view of the data. **A.** UMAP of combined samples with specific, low-level, cell type annotations. **B., C.,** and **D.** Subset and reprojection consisting of immune cells, endothelial cells, and fibroblasts. **E.** The proportion of each low-level cell type inclusive of all samples. Note that the y-axis is in log scale. **F.** Stacked violin plot of marker gene expression by low-level cell type annotation.

We subsequently refined our cell type annotation by subclustering the immune cell, fibroblast, endothelial cell, and adipocyte populations (Figure 2A – 2F). The immune cell cluster separated into clearly defined groups of lymphocytes and myeloid cells. The lymphocytes are made up of T cells (*Skap1+*, 0.92%), B cells (*Pax5*+, 0.37%), and natural killer cells (*Gzmk+*, 0.34%). The myeloid cells (*Itgam+*) consist of neutrophils (*S100a9+*, 0.22%), monocytes (*Fn1+*, 0.02%), M2-macrophages (*Cd163*+, 1.95%), M1-macrophages (*Lyz2*+, 0.45%), and dendritic cells (*Flt3+*, 0.14%) (Figure 2B). The fibroblast population is comprised of four groups based on the expression of the genes *Bmper* and *Pi16*; similar to a study by Burl et.al^21^. Those four groups were labelled Fibroblasts_Bmper+ (1.5%), Fibroblasts_Bmper+_Nrxn1+ (4.9%), Fibroblasts_Pi16+ (1.7%), and Fibroblasts_Pi16++ (1.1%) (Figure 2C). Endothelial cells were also represented by several subtypes (Figure 2D). Of note, we found groups of endothelial cells expressing marker genes that have previously been associated with lymphatic (*Prox1*+, 1.5%), venous (*Vwf*+*Nr2f2+*, 1.9%), arterial (*Fbln5*+, 3.2%) and capillary (*Krp*+, 9.3%) endothelial cells^22,23^. Finally, we subset the adipocytes and found a large group of brown adipocytes marked by their expression of *Ucp1* that makes up 57.4% of the cell population (Figure 2E). There were also two groups of *Pparg+Ucp1*- adipocytes that both express *Car3* but only one group expressed *Kcnip1*. The *Car3+Kcnip1+* cluster and the *Car3+Kcnip-* cluster make up 4.3% and 2.5% of the cell population respectively. These groups may mark white or beige adipocytes, or they may be committed adipocyte progenitor cells (APCs)^21,24^. The overall expression pattern of key marker genes showed clear segregation by cell type (Figure 2F).

### Cell type proportions vary by diet, diet duration, and sex

The combined dataset with final cell type annotations was split by sample into 25 smaller datasets. Within each of the individual sample datasets, cell type proportions were calculated by taking the number of cells of a particular type and dividing by the total number of cells. Those samples were then grouped by treatment and cell type proportions were compared. A two-tailed Welch’s t-test was used to calculate significant differences in the cell type proportions across each treatment.

**Figure 3:**
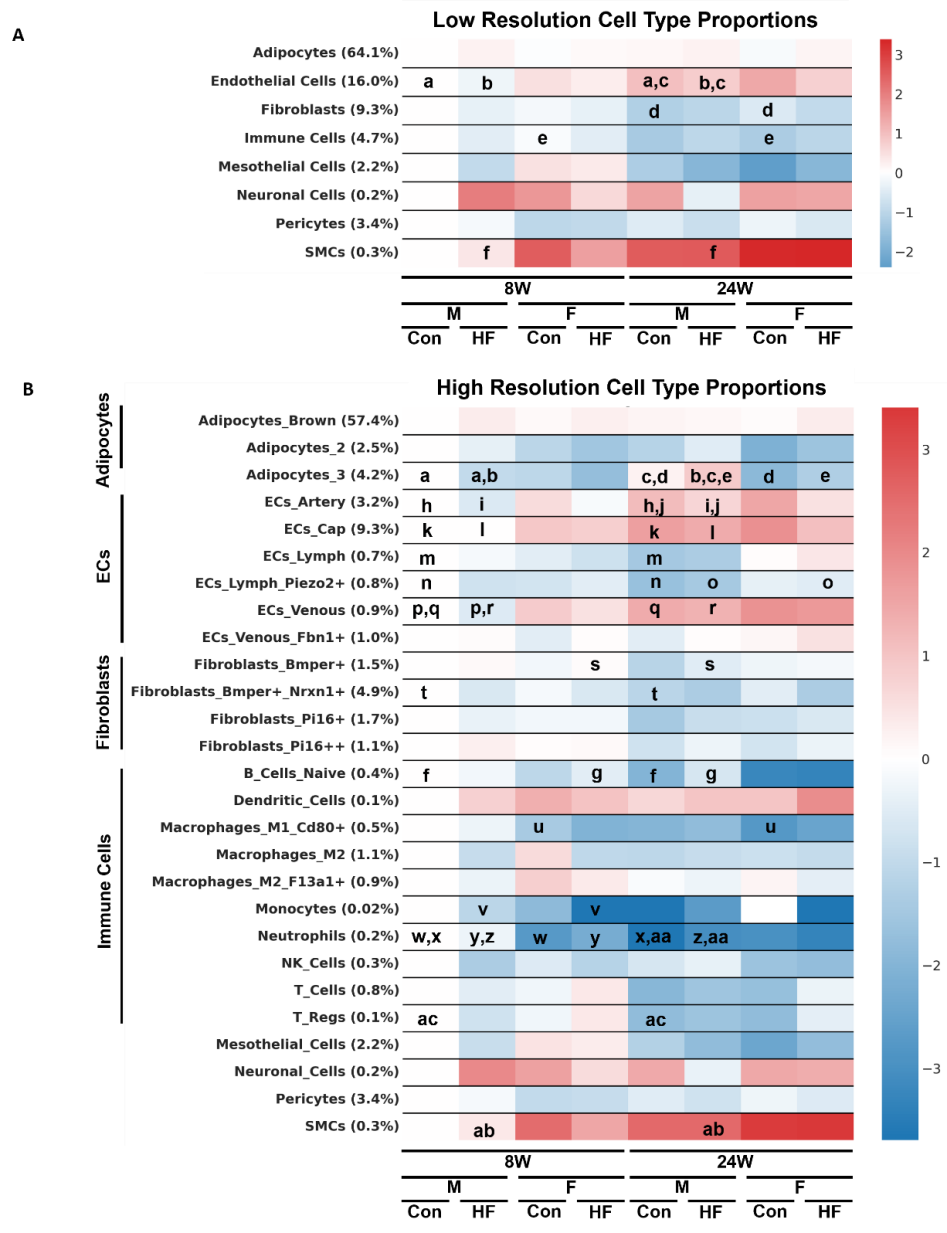
Heatmaps representing the proportions of **(A)** low resolution and **(B)** high resolution cell types and how they differ across each treatment category. The y-ticks are labeled with the cell types and their mean proportion across all treatment categories. The color bar legend indicates the log_2_(fold change) of the treatment proportion relative to the 8-week, control-diet fed, male rat treatment group for each cell type. All statistical comparisons were made using a two-tailed Welch’s t-test. Significant differences (p-value ≤ 0.05) between treatments are marked by the letters on the plot. I.e. “a” means that there is a significant difference in the proportion of endothelial cells between diet durations in male rats fed a control diet.

The proportions of cell types differed between groups, especially when compared across time on different diets (Figure 3A). We began by only considering the eight low-resolution cell types. Between HF and control diets, only endothelial cells showed differences in abundance. There was a significantly lower proportion of endothelial cells in the male rats fed a HF versus control diet for 24 weeks. Between sexes, there was a significantly higher proportion of fibroblasts in the female versus male rats fed a control diet for 24 weeks. This difference was not observed in the HF diet fed rats. Comparing the 8 and 24 week time points, we found a significantly higher proportion of endothelial cells was present at 24 weeks in male rats fed either a HF or control diet, a lower population of immune cells at 24 weeks in female rats fed a control diet, and a higher population of SMCs at 24 weeks in male rats fed a control diet.

We then tested cell type proportion differences in the high-resolution cell types and again found most differences to be across diet durations (Figure 3B). When comparing the HF-diet-fed rats to the control diet-fed rats, we found a smaller proportion of arterial ECs in male rats at 24 weeks, a lower proportion of venous ECs in male rats at 8 weeks, a smaller proportion of *Kcnip1+* adipocytes in male rats at 8-weeks, a higher proportion of *Kcnip1+* adipocytes in male rats at 24 weeks, and a higher proportion of neutrophils in male rats at 24 weeks. When comparing the female rats to the male rats, the female rats were found to have: a lower proportions of *Kcnip1+* adipocytes at 24 weeks on both HF and control diets, a higher proportion of *Bmper+* fibroblasts at 24 weeks on control diet, a higher proportion of *Piezo2+* lymphatic ECs at 24 weeks on HF diet, a lower proportions of neutrophils at 8 weeks in both HF and control diets, a lower proportion of B cells at 24 weeks in the HF diet, and a lower proportion of monocytes at 8 weeks on the HF diet. Finally, when comparing the 24 week on diet data to the 8 week on diet data, we found that at 24-weeks there was: a lower proportion of *Bmper+Nrxn1+* Fibroblasts in male rats fed a HF diet, a lower proportion of lymphatic ECs in male rats fed a control diet, a lower proportion of *Piezo2+* lymphatic ECs in male rats fed a control diet, a greater proportion of arterial ECs in male rats fed either a HF or a control diet. At 24 weeks there was also a higher proportion of capillary ECs in male rats fed a HF or control diet, a higher proportion of venous ECs in male rats fed either a HF or control diet, a greater proportion of *Kcnip1+* adipocytes in male rats fed a HF diet, a higher proportion of SMCs in male rats fed a HF diet, a lower proportion of Neutrophils in male rats fed either a HF or control diet, a lower proportion of *Cd80+* M1-Macrophages in female rats fed a control diet, a lower proportion of B cells in female rats fed a control diet, and a lower proportion of regulatory T cells in male rats fed a control diet.

### Cell type specific gene expression patterns are affected by diet, diet duration, and sex

**Extended Data Figure 2:**
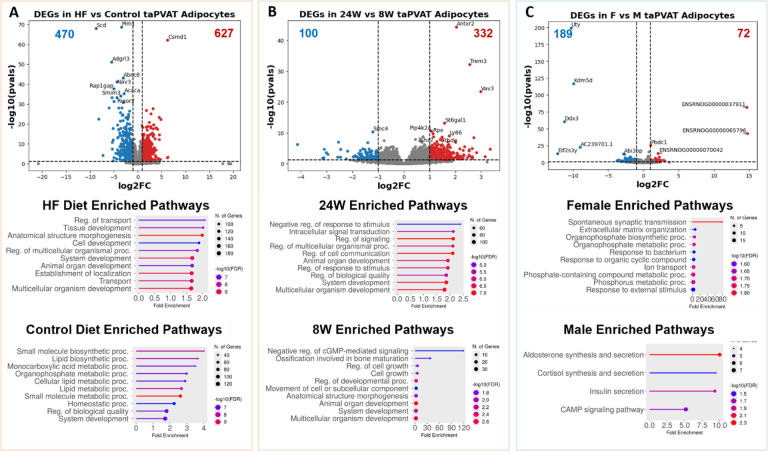
**A.** (Top) Volcano plot of differentially expressed genes (DEGs) in adipocytes between high fat and control diets. (Middle, Bottom) Enriched GO biological process pathways in rats fed a HF or control diet (includes 8-week, 24-week, M and F data). **B.** (Top) Volcano plot of DEGs between 8W and 24W diet durations. (Middle, Bottom) Enriched GO biological process pathways in rats on diet for 24-weeks or 8-weeks (includes control diet, HF diet, M and F data). **C.** (Top) Volcano plot of DEGs between female and male rats. (Middle, Bottom) Enriched GO biological process pathways in female or male rats (includes control diet, HF diet, 8-week, and 24-week data).

We investigated the effect of three variables (diet, diet duration, and sex) across cell type on gene expression levels. We first studied gene expression differences across the whole tissue, inclusive of all cell types. When comparing all the control diet samples to all the HF diet samples (this includes 8-week, 24-week, male and female data), we found a total of 577 differentially expressed genes (DEGs) (Extended Data Figure 2A top panel). The 304 upregulated DEGs in the HF diet were associated with carboxylic acid catabolic process and ion transport pathways (Extended Data Figure 2A middle panel), whereas the 273 downregulated genes were linked to broad fatty acid metabolism (Extended Data Figure 2A bottom panel). Similarly, when we compared the 8-week timepoint samples to the 24-week timepoint samples, we found 784 DEGs (Extended Data Figure 2B top panel). The 258 upregulated genes at 24 weeks were largely associated with regulation of cell motility (Extended Data Figure 2B middle panel), and the 526 downregulated genes were linked to immune responses (Extended Data Figure 2B bottom panel). Lastly, when we compared the male rat samples to the female rat samples, we found 181 total DEGs (Extended Data Figure 2C top panel). The 43 upregulated genes in female rats were associated with positive regulation of vesicle fusion pathways (Extended Data Figure 2C middle panel), whereas the 138 downregulated genes were linked to leukocyte migration (Extended Data Figure 2C bottom panel).

When separating treatment groups by time on diet the number of DEGs in male rats fed a HF versus control diet rises from 266 at 8-weeks on diet to 451 at 24 weeks on diet. However, the number of DEGs in female rats fed a HF versus control diet falls from 554 at 8-weeks to 238 at 24-weeks. There are 83 DEGs conserved across the four treatment groups (Extended Data Figure 2D). When comparing the 8- and 24-week time points, there are again more DEGs in the male rats than the female rats. The number of DEGs in both males and females is higher in control diet fed rats than in HF diet fed rats. There are only two DEGs conserved across the four treatment groups considering diet duration as a variable (Extended Data Figure 2E). The fewest number of DEGs occurs when comparing female to male rats. The HF diet fed rats again had fewer DEGs than the control diet fed rats. The number of DEGs also increased in the 24-week on-diet group compared to the 8-week on-diet group. There were only three DEGs shared among all treatment groups (Extended Data Figure 2F).

**Extended Data Figure 3:**
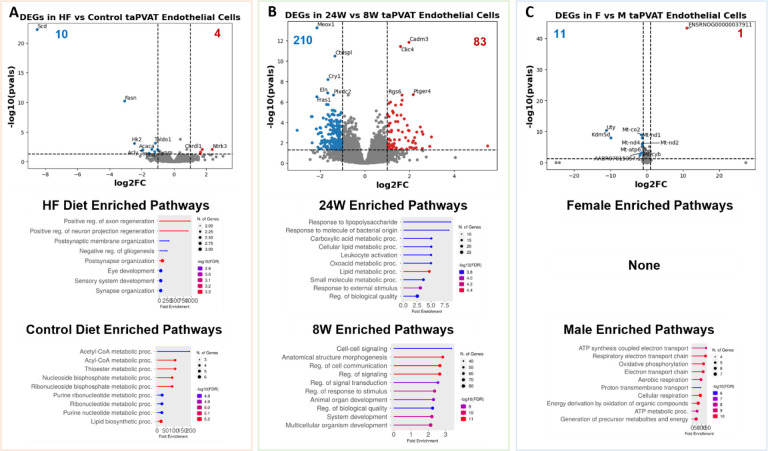
**A.** (Top) Volcano plot of differentially expressed genes (DEGs) in endothelial cells between high fat and control diets. (Middle, Bottom) Enriched GO biological process pathways in rats fed a HF or control diet (includes 8-week, 24-week, M and F data). **B.** (Top) Volcano plot of DEGs between 8W and 24W diet durations. (Middle, Bottom) Enriched GO biological process pathways in rats on diet for 24-weeks or 8-weeks (includes control diet, HF diet, M and F data). **C.** (Top) Volcano plot of DEGs between female and male rats. (Middle, Bottom) Enriched GO biological process pathways in female or male rats (includes control diet, HF diet, 8-week, and 24-week data).

**Extended Data Figure 4:**
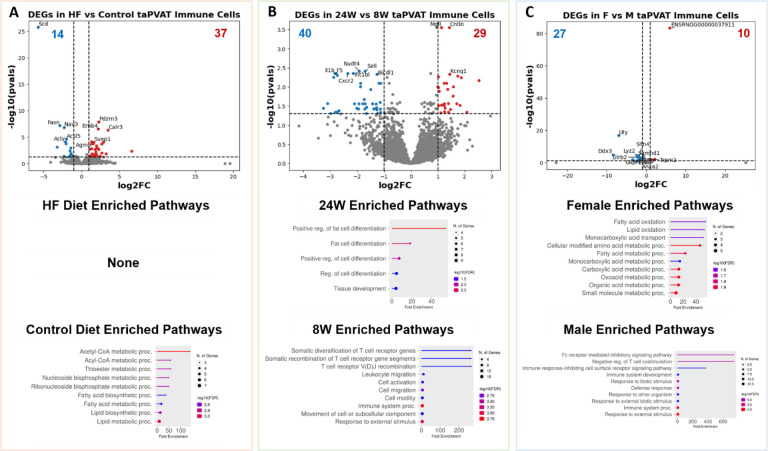
**A.** (Top) Volcano plot of differentially expressed genes (DEGs) in immune cells between high fat and control diets. (Middle, Bottom) Enriched GO biological process pathways in rats fed a HF or control diet (includes 8-week, 24-week, M and F data). **B.** (Top) Volcano plot of DEGs between 8W and 24W diet durations. (Middle, Bottom) Enriched GO biological process pathways in rats on diet for 24-weeks or 8-weeks (includes control diet, HF diet, M and F data). **C.** (Top) Volcano plot of DEGs between female and male rats. (Middle, Bottom) Enriched GO biological process pathways in female or male rats (includes control diet, HF diet, 8-week, and 24-week data).

**Figure 4:**
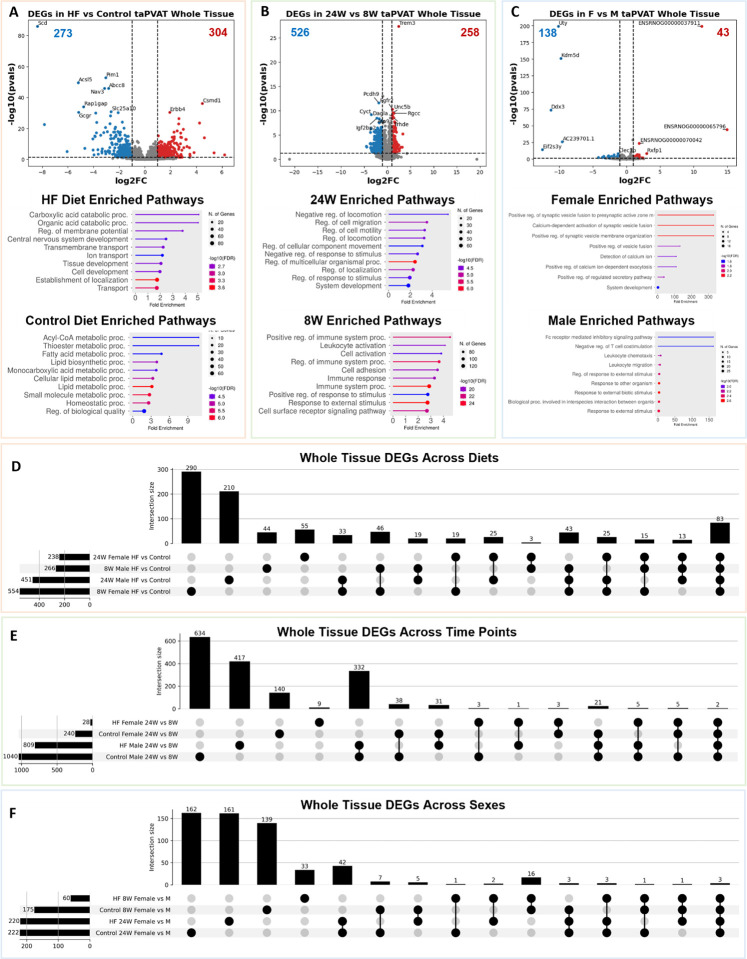
**A.** (Top) Volcano plot of differentially expressed genes (DEGs) between high fat and control diets. (Middle, Bottom) Enriched pathways in rats fed a HF or control diet (includes 8-week, 24-week, M and F data). **B.** (Top) Volcano plot of differentially expressed genes (DEGs) between 8W and 24W diet durations. (Middle, Bottom) Enriched pathways in rats on diet for 24-weeks or 8-weeks (includes control diet, HF diet, M and F data). **C.** (Top) Volcano plot of differentially expressed genes (DEGs) between female and male rats. (Middle, Bottom) Enriched pathways in female or male rats (includes control diet, HF diet, 8-week, and 24-week data). **D.** Upset plot of DEGs across treatments in the whole tissue when comparing the control diet data to the HF diet data. There were 83 DEGs that were found in all 4 comparisons. **E.** Upset plot of DEGs across treatments in the whole tissue when comparing the 8-week diet duration to the 24-week diet duration. **F.** Upset plot of DEGs across treatments in the whole tissue when comparing the male and female rats.

Building on the whole-tissue analyses, we conducted a cell-type specific differential gene expression analysis to dissect how each cell type individually responds to diet. This analysis highlighted cell-type-specific DEGs and enriched pathways that are otherwise masked in whole-tissue comparisons. When considering only the eight low-resolution cell types, adipocytes displayed the most pronounced gene expression changes across all three variables: diet, time on diet, and sex (Figure 4A–C). The close overlap between adipocyte DEGs and whole-tissue DEGs implies that adipocytes are a primary driver of the observed whole-tissue gene expression changes. In adipocytes in response to a HF diet, upregulated DEGs were significantly enriched in tissue development and transport pathways. When comparing diets, downregulated genes in adipocytes were associated with broad fatty acid metabolism. After 24 weeks of diet exposure, adipocyte upregulated genes were linked to pathways involved in cell signaling and communication, while downregulated genes were associated with cell growth. Sex-specific analyses revealed enrichment for hormone synthesis and secretion pathways in males, whereas upregulated DEGs in females were associated with phosphorus metabolism. Endothelial cells also demonstrated notable gene expression changes, particularly in response to the duration of diet (Extended Data Figure 3A–C). In 24-week versus 8-week diet comparisons, upregulated genes in endothelial cells were involved in fatty acid metabolism pathways and leukocyte activation. The downregulated genes were largely associated with cell-cell signaling pathways. This suggests a potential reduction in endothelial signaling with aging or prolonged dietary stress, coinciding with increased metabolic and inflammatory demands. Immune cells exhibited fewer DEGs across all three variables (Extended Data Figure 4A–C).

**Extended Data Figure 5:**
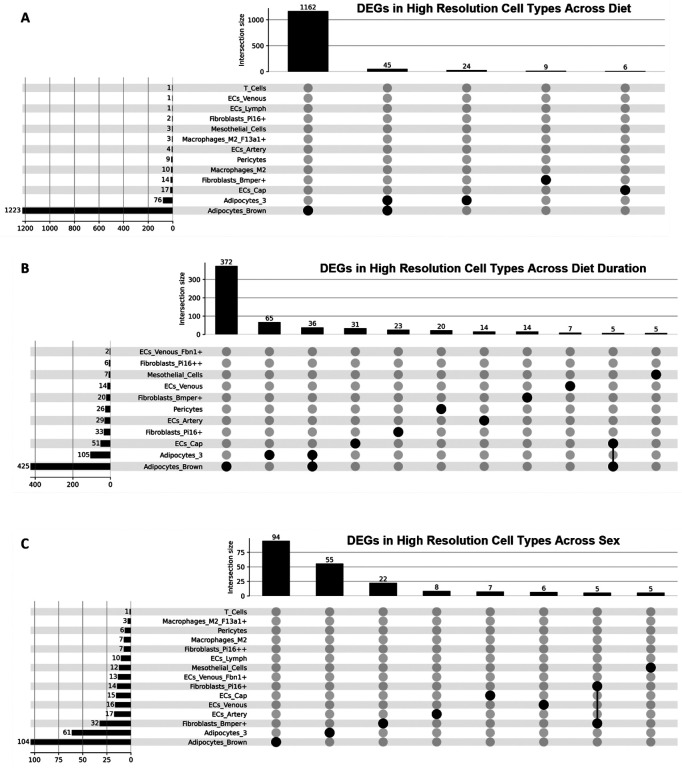
Upset plots of DEGs in high-resolution cell types across **(A)** diet, **(B)** diet duration, and **(C)** sex.

Like adipocytes and endothelial cells, genes downregulated in response to the HF diet in immune cells were significantly associated with broad fatty acid metabolism pathways. At 24 weeks on diet, upregulated genes were associated with fat cell differentiation, whereas the upregulated genes at 8 weeks were associated with somatic diversification and recombination of T-cell receptor genes. Female rats were highly enriched for fatty acid metabolism pathways, and male rats were enriched for immune cell response inhibition pathways. Pericytes, mesothelial cells, neuronal cells, and SMCs had low numbers of DEGs across all three variables. Lastly, when we consider the 28 high-resolution cell types, brown adipocytes had the highest number of DEGs (Extended Data Figure 5A–C). These findings collectively underscore the value of single-cell transcriptomics in uncovering cell-type-specific responses, which are otherwise masked in bulk tissue analyses.

### Cell-cell Communication analysis reveals alterations and heterogeneity in signaling between and within cell types

Given the multicellular composition of PVAT and its putative role in regulating vascular function in health and disease, we wanted to examine the complex web of cell-cell communication among the varied cell types in PVAT. For our preliminary analysis of communication among cell types in aggregate, we used NicheNet^25^, which uses single-cell gene expression and a prior model that incorporates intracellular signaling to characterize how ligands from a “sender” cell type functionally communicate with a “receiver” cell type, as reflected in induction of downstream target genes. We also used CellChat^26^ and CellPhoneDB^27^ within the LIANA^28^ framework to predict key ligand-receptor interactions driving condition-specific cellular responses.

There were broad similarities in inferred signaling pathways across sex and diet (Table 1). For example, vascular endothelial growth factor (VEGF) - Neuropilin-1 (NRP1) signaling (blue shading, Table 1) from adipocytes to endothelial cells was one of the top pathways in control male, control female, and HF female animals, but was absent in HF males. This is notable, given the known role of the VEGF - NRP1 pathway in regulation of angiogenesis, and vascular remodeling and permeability^29^. Conversely, SORBS1 – ITGA1 signaling (green shading, Table 1) from adipocytes to endothelial cells was one of the top interactions only in 24-week HF males. SORBS proteins are implicated in stiffness-sensing and contractile force generation^30^, suggesting an enhanced mechanical signal transduction role for adipocytes in PVAT under high-fat diet conditions. Likewise, multiple Thrombospondin-1 (THBS1) - integrin signaling pathways, which are involved in mechanotransduction, angiogenesis, and vascular remodeling^31,32^, were prominent in HF but not control 8-week males (Extended Data Table 1).

**Figure 5:**
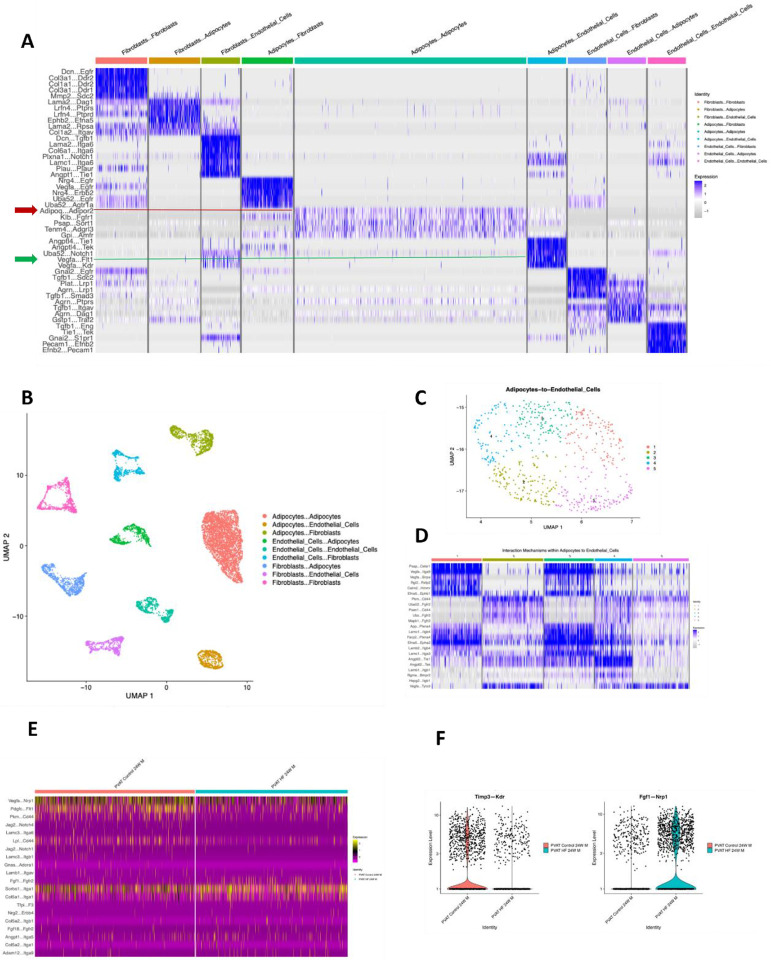
Cell-Cell Communication in PVAT Using NICHES. **A.** Mapping of cell-cell interactions in PVAT from 8-week control males. **B.** UMAP visualizations highlight distinct pairwise clusters of cell-cell interactions and communication patterns. **C**. UMAP of adipocyte-to-endothelial cell interactions in 8-week control males. **D.** Heatmap of ligand-receptor interactions between adipocytes and endothelial cells in 8-week control male, emphasizing key signaling pathways. **E.** Comparison of top ligand-receptor interactions between adipocytes and endothelial cells in 24-week control and high-fat diet (HF) males. **F.** Violin plots of **Timp3—Kdr** and **Fgf1—Nrp1**, showing increased activity in control conditions from 8 to 24 weeks and elevated expression in HF males over time.

While the tools utilized above all infer interactions on a cell-population level, we wanted to delve deeper to investigate heterogeneity in signaling mechanisms within and between cell types at the level of individual cells. For this purpose, we utilized NICHES, a tool that infers cell-cell communication at a truly single-cell level based on simultaneous expression of ligands in the sending cell and cognate receptors in the receiving cell^33^. In control 8-week males, NICHES revealed distinct modules of signaling pathways involved in communication between specific sender and receiver cell types at single-cell level, with few interactions common to signaling among different cell type pairs (Figure 5A). For example, Adiponectin (ADIPOQ) to Adiponectin receptor ADIPOR2 signaling is largely restricted to adipocyte – adipocyte communication (Figure 5A, red arrow and red horizontal line), while VEGF - vascular endothelial growth factor receptor 1 (FLT1) signaling was found in communication from both adipocytes and fibroblasts to endothelial cells (Figure 5A, green arrow and green horizontal line). Using NICHES, we also projected individual cell-pair signaling events onto a 2-dimensional ligand-receptor interaction space, revealing distinct clusters of signaling events between cell-type pairs (Figure 5B). Zooming in to examine only adipocyte to endothelial cell communication, we found considerable heterogeneity in signaling mechanisms (pathways) between individual cell pairs, as reflected in distinct clusters of signaling events (Figures 5C and 5D). Further, the occurrence of signaling pathways varied between control and HF diet (Figure 5E). For instance, TIMP3 – KDR signaling was less frequent and FGF1 – NRP1 signaling more frequent when comparing 24-week HF to 24-week control diet males (Figure 5F).

### RNA velocity analysis reveals both gradual and abrupt transitions in gene expression dynamics according to time on HF diet

**Figure 6:**
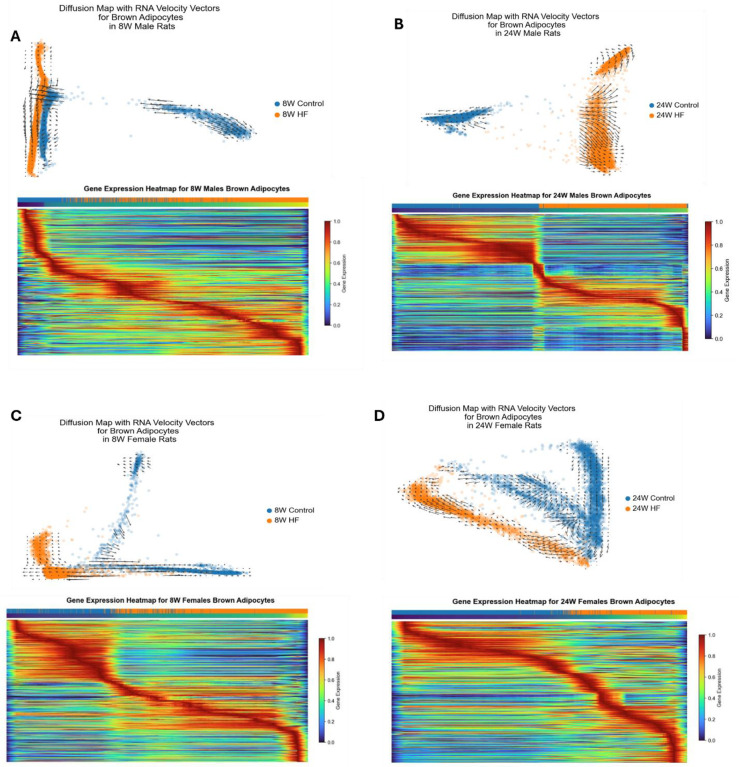
RNA velocity and gene expression heatmaps for brown adipocytes using scVelo. **A.** 8-week control males versus 8-week high-fat males. The top panel shows RNA velocity vectors in a diffusion map embedding. The velocity vectors indicate the likely shift in gene expression states in nearby cells. Longer vectors indicate a greater RNA velocity (i.e., greater shift in gene expression space). The bottom panel depicts a heatmap displaying the gene expression magnitude of the top 500 genes driving the latent time trajectory. Red indicates high expression, while blue indicates low expression. Rows represent individual genes and columns correspond to cells, color coded by diet (blue for control and orange for high-fat). The progression through latent (pseudo) time is shown from purple (start) to yellow (end). **B.** 24-week control males versus 24-week high-fat males. **C.** 8-week control females versus 8-week high-fat females. **D.** 24-week control females versus 24-week high-fat females.

Next, we investigated gene expression dynamics and gene regulatory networks within brown adipocytes, the predominant cell type in taPVAT. Specifically, we used scVelo^34^ to reveal transcriptional dynamics and progression of unique cell states in response to a high-fat diet through generalization of cellular RNA velocity^35^. The velocity was calculated for each gene in each cell, following which the computed RNA velocity vectors were embedded in a 2-dimensional diffusion map visualization for each group of brown adipocytes (Figures 6A–D, **top panels**). These visualizations thus depict cell transition dynamics in brown adipocytes induced by diet in both male and female animals. In addition, heat maps displaying the change in gene expression from control to HF diet through latent time (pseudotime) progression were generated from the scVelo workflow (Figures 6A–D, **bottom panels**). We chose to display changes in gene expression for the top 500 most likely genes driving the latent time trajectory from control to HF diet in both male and female rats at 8 weeks (Figures 6A and 6C) and 24 weeks (Figures 6B and 6D).

At 8 weeks, the RNA velocity vectors show a strong directionality from control to HF diet associated cell states in both males (Figure 6A, top panel) and females (Figure 6C, top panel), but little-to-no such directionality in the 24-week groups (Figures 6B and 6D, top panels). The heat maps corroborate this, with the 8-week comparisons (Figures 6A and 6C, bottom panels) showing a smooth transition in gene expression states from control diet to high-fat diet, and throughout pseudotime. However, the 24-week comparisons (Figures 6B and 6D, bottom panels) show a sharp step off in gene expression between the control and high-fat diet cells suggesting a more abrupt state transition. This aligns with expectations because, after 24 weeks on a given diet, we assume cells have had sufficient time to “settle into” their respective gene expression states given their environment. This leads to a greater divide between 24-week control and 24-week high-fat gene expression states compared to 8 weeks.

### Gene Regulatory Network Analysis identifies key transcriptional drivers of adipocyte state transition induced by HF diet

To identify key transcription factors driving the shift of adipocytes from control towards a high-fat diet transcriptomic state for both male and female rats, we used CellOracle^14^ to explore differential gene regulatory networks active in each case. Specifically, we looked at which transcription factors undergo the highest functional change from control to high-fat diet conditions. Using CellOracle, we computed the centrality or importance of each transcription factor in the condition-specific network via several alternative metrics. These centrality measures are calculated within CellOracle’s workflow and include eigenvector centrality, degree centrality out, and betweenness centrality. Higher eigenvector centrality measures characterize transcription factors that have more interactions with highly connected genes^36^. Degree-centrality-out provides information about the number of genes a transcription factor regulates, while betweenness centrality gives an indication of which transcription factors act as a bottleneck between different highly connected sub-networks within a gene regulatory network^36–38^. Taking these three centrality measures together, we hypothesized that transcription factors that have a high score in each measure may be important for overall regulatory control in a specific condition.

The ratio of each of the centrality measures in HF vs. control diet provides a measure of which transcription factors become increasingly more functionally important for each group of adipocytes. Higher ratios mean that the transcription factor gained more importance in that specific centrality measure in the high-fat diet group compared to the control-diet. For each centrality ratio, the transcription factors were ranked by magnitude (highest to lowest), with the highest ratio (top rank) receiving a rank of 0 and subsequent TFs receiving rank scores of 1, 2, and so on. Each transcription factor’s “total score” was determined by summing the ranks from each measure. For example, in adipocytes from female animals at 24–weeks, the top ranked transcription factor was *Nr4a1* with a total ranked score of 3 due to its eigenvector, degree centrality out, and betweenness centrality ranks of 0, 1, and 2, respectively (Extended Data Table 2).

**Figure 7.**
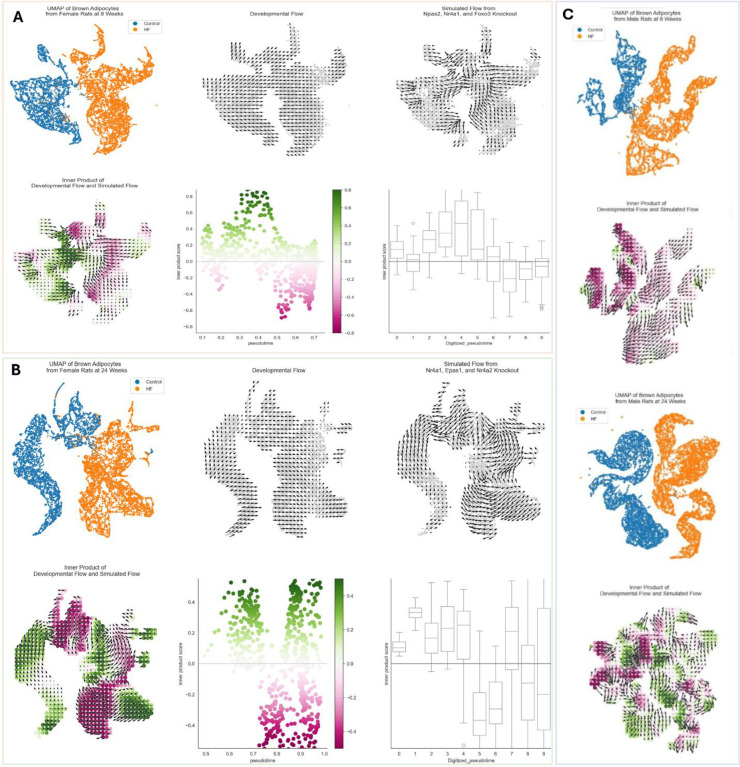
Overview of CellOracle workflow and *in-silico* TF knockout in brown adipocytes. **A.** Panel A (top) depicts results from 8-week females (control versus high-fat diets). **B.** Panel B (bottom) depicts results from 24-week females (control versus high-fat diets). Top left: UMAP plot displaying control cells (blue) and high-fat diet cells (orange). Top center: Developmental flow vectors suggest a baseline progression from control cells to high-fat diet cells in gene expression space. Top right: Simulation vectors show the predicted transition in cell states after transcription factor knockout, representing the altered likelihood of a cell state change post-knockout. Bottom left: The dot product of developmental flow and simulation vectors, where green indicates a positive dot product, suggesting stabilization of the respective gene expression states, and pink indicates a negative dot product, implying resistance to transitions to these states. Bottom center: Scatter plot with each point representing a cell, colored by its dot product score. The x-axis represents pseudotime, and the y-axis quantifies the dot product score, illustrating gene expression state evolution over pseudotime. Bottom right: Box and whisker plots summarizing the data by binning cells into 10 groups across pseudotime, showing the distribution of dot product scores within each bin. **C.** Panel C depicts results between control and high-fat diets from 8-week males (left) and 24-week males (right). The top subfigures are UMAP plots displaying control cells (blue) and high-fat diet cells (orange). The bottom subfigures are the dot product of developmental flow and simulation vectors, where green indicates a positive dot product, suggesting stabilization of the respective gene expression states, and pink indicates a negative dot product, implying resistance to transitions to these states.

To validate the significance of these inferred transcription factors, we performed *in-silico* perturbation experiments using CellOracle. For each comparison, the top three transcription factors, as determined by the combined centrality measurement scores (see Methods) were computationally knocked out from the gene regulatory network. This is depicted with a 2D UMAP embedding of adipocytes from 8-week females (Figure 7A, upper panels) with knockout of *Npas2*, *Nr4a1*, and *Foxo3*, 24-week females (Figure 7B, upper panels) with knockout of *Nr4a1*, *Epas1*, and *Nr4a2*, 8-week males (Figure 7C, left panels, and Extended Data Figure 8) with knockout of *Hlx*, *Bhlhe40*, and *Ezh2*, and in 24-week males (Figure 7C, right panels, and Extended Data Figure 8) with knockout of *Cebpb*, *Nr4a3*, and *Nr4a2*.

This section focuses on the female analysis for clarity and brevity, but the full results for males are included in Extended Data Figure 8. The “developmental / pseudotime vectors” (small arrows in Figures 7A and 7B, upper middle panels) show the direction of diet-induced transition, while the “perturbation / knockout vectors” (small arrows in Figures 7A and 7B, upper right panels) point in the direction of the most likely transition after simulated transcription factor knockout. The perturbation vectors demonstrate a uniform trajectory *away* from the high-fat diet-associated cell states, especially in the 8-week females. This is shown by a “perturbation score” (Figures 7A and 7B, lower panels) which is the inner-product value between the pseudotime vectors and the knockout vectors. Green colors represent regions where the pseudotime vectors and perturbation vectors align in the same direction, such that these cell states become more stabilized. Pink colors represent the opposite, where differentiation and perturbation vectors are aligned in opposite directions, thus representing cell states that are resisted or suppressed post-knockout. Effectively, simulated knockout disrupts the progression from control to high-fat diet associated cell states, even reversing it in some sections along the pseudotime trajectory (Figures 7A and 7B, lower middle and right panels).

## DISCUSSION

This study provides a single-nucleus analysis of thoracic aortic perivascular adipose tissue (taPVAT) in Dahl SS rats, providing key insights into how diet, sex, and diet duration shape cell type composition and cell-type specific gene expression. Our identification of eight major cell types in taPVAT, dominated by adipocytes, endothelial cells, and fibroblasts, mirrors prior characterizations of adipose tissue^21,24^. However, the ability to further resolve these populations into twenty-eight high-resolution subtypes, such as dendritic cells and lymphatic endothelial cells, offers novel insights. Notably, the predominance of brown adipocytes (57.4%) in this tissue may reflect its specialized role in thermogenesis and metabolic regulation. Cell type composition changes highlighted significant differences influenced by diet, diet duration, and sex. For instance, HF diet-fed male rats exhibited a reduction in endothelial cells, aligning with evidence of endothelial dysfunction under metabolic stress^39,40^. Sex-specific differences in fibroblast populations, including a higher proportion of *Bmper*+ fibroblasts in females, suggest potential sex-specific remodeling of extracellular matrix dynamics, which could influence vascular integrity and stiffness.

Differential gene expression analyses provided additional mechanistic insights. Adipocytes emerged as the primary driver of whole-tissue transcriptional changes, with upregulated genes enriched in pathways related to tissue development and transport under HF diet conditions. This observation suggests that adipocytes play a central role in adapting to metabolic challenges, likely through increased lipid storage and altered cell signaling. Endothelial cells also displayed significant gene expression changes, particularly with prolonged dietary exposure, highlighting their role in vascular inflammation and metabolic adaptation.

Our analysis of cell-cell communication revealed extensive HF-diet induced alterations in ligand-receptor interactions between cell type pairs and signaling networks that may contribute to vascular dysfunction in hypertension. We identified distinct patterns of signaling pathways influenced by sex and diet. VEGF-NRP1 signaling, linked to angiogenesis and vascular remodeling, was induced in high-fat females but repressed in high-fat males, suggesting sex-specific vascular remodeling differences. In contrast, SORBS1-ITGA1 signaling emerged in high-fat males, indicating enhanced mechanotransduction under dietary stress. ADIPOQ-ADIPOR2 signaling was limited to adipocyte autocrine communication, while VEGF-FLT1 facilitated adipocyte and fibroblast interactions with endothelial cells, highlighting diverse signaling modules in PVAT. Pathway-specific changes showed reduced TIMP3-KDR signaling, linked to extracellular matrix remodeling, and increased FGF1-NRP1 signaling under high-fat conditions, reflecting shifts in remodeling processes. Clustering of ligand-receptor interactions at the level of individual cells revealed considerable heterogeneity of signaling even within the same cell-type pair, and distinct modules regulating localized vascular responses.

We should note that the varied cell-cell computational tools we used often make different assumptions about ligand-receptor activity based on gene expression, which may not always correlate with functional signaling activity. The discrepancies between tools like NicheNet, NICHES, and LIANA due to differing algorithms and databases highlight the need for cross-validation and experimental validation of predictions. The spatial organization of cells within PVAT, which can influence signaling patterns, was not directly assessed in this study. Validation of predicted signaling pathways using functional assays, such as ligand-receptor binding studies, would improve biological relevance. This study reveals high-fat diet-induced changes in PVAT signaling, uncovering mechanisms of vascular dysfunction and potential targets for mitigating PVAT-driven pathologies.

One can imagine a “typical” brown adipocyte that expresses a distribution of genes within some normal limit. This distribution of expected genes is likely to change in response to a perturbation, or more specifically in this study, a dietary perturbation. We asked the question, in what way does this distribution change, gradually or in a sudden shift? In both male and female rats, the distribution of gene expression states of brown adipocytes along transition trajectories from the PVAT of control- or HF-diet fed rats for 8-weeks overlapped more than their respective 24-week gene expression states. This resulted in gene expression states that appear to shift gradually at the 8-week time point, but show a sharp step-off at 24 weeks. Likewise, RNA velocity analyses demonstrated a more uniform “flow” from control to HF cell states in the 8-week group compared to the 24-week groups.

Together, this suggests that the shift in gene expression in response to high-fat diet is not only gradual, but begins to take place at 8 weeks. If the change in gene expression were to occur later than 8-weeks, the gene expression between groups would most likely appear more “flat-lined” on the 8-week heatmaps, i.e., there is not much difference in the distributions of gene expression states between groups at this time point. On the other hand, if the shift in gene expression were to have occurred before 8-weeks, the likely observation would be a sharp drop-off between groups, indicating a shift in gene expression had already taken place and the cells have “settled in” to their new gene expression states. This is what we see in the 24-week group comparisons, especially in the male rats.

**Extended Data Figure 6:**
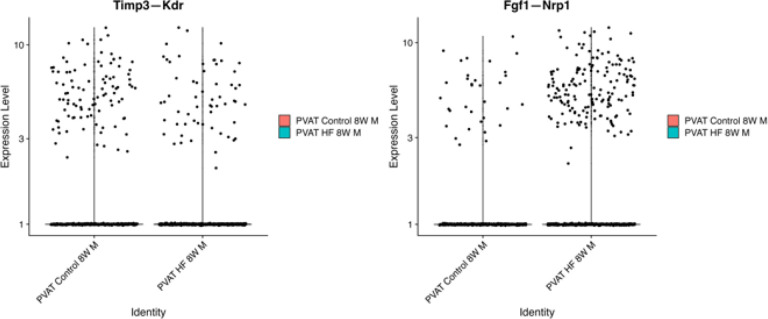
Violin plots of Timp3—Kdr showing increased control activity and Fgf1—Nrp1 with elevated HF activity. Activity rises from 8 to 24 weeks, reflecting progression with prolonged HF diet.

To better understand the full progression from health (8-week control) to HF diet-associated hypertension (24-week high-fat), we analyzed brown adipocytes from 8-week control diet, 8-week high-fat diet, and 24-week high-fat diet simultaneously. We included brown adipocytes from both male and female rats together to maximize the number of cells. We observed a gradual gene expression transition from 8-week control to 24-week high-fat diet that was independent of age, with corroborating RNA velocity analysis (Extended Data Figures 6A and 6B), suggesting a slow shift in gene expression throughout this process.

**Extended Data Figure 7:**
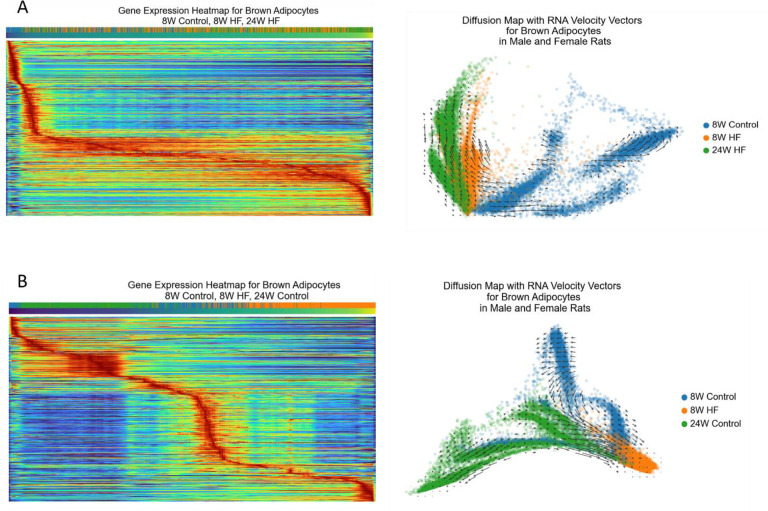
scVelo RNA velocity and heat map plots showing full pseudotime trajectory in combined male and female brown adipocytes. **A.** Gene expression trajectory through pseudotime from 8-week control to 8-week high-fat to 24-week high-fat groups. **B.** Gene expression trajectory through pseudotime from 8-week control to 8-week high-fat to 24-week control. There is a smooth trajectory in gene expression (heat map) and a uniform flow (RNA velocity plot) from 8-week control through 24-week high-fat (panel A) that is lost when swapping to 24-week control (panel B). This implies that this result is not simply due to age differences in the animals, and is instead suggests a steady progression from control-like cell states to high-fat diet-associated cell states.

Our analysis of gene regulatory networks identified receptors of the nuclear receptor subfamily Nr4a (*Nr4a1*, *Nr4a2*, and *Nr4a3*) among the top three most functionally important transcription factors in every subgroup of rats except for 8-week males. In the latter group, these receptors were found among the top 20 most important factors (Extended Data Tables 2 and 3). This suggests that the Nr4a subfamily is a notable transcriptional regulator in PVAT of high-fat-diet associated hypertension and vascular dysfunction. Nra4 nuclear receptors are orphan receptors, and growing evidence implicates them in having an influential role in cardiovascular disease^41^. A reduction in adipogenic capacity is a major contributor to adipocyte hypertrophy which in turn drives several deleterious pathways and promotes inflammation^42^. Given that at least one member of the Nr4a subfamily is implicated in inhibiting adipogenesis^43^, these transcription factors could be potential drug targets for addressing obesity-related hypertension. This possibility is supported by the computational knockout experiments (Figure 7 and Extended Data Figure 7A and 7B) that demonstrated a strong resistance, or even reversal, of high-fat diet associated cell states. One limitation of these analyses, however, is that we do not know which specific promoters are accessible in this context due to the lack of single-nucleus chromatin accessibility data, which would improve identification of target gene-specific upstream transcriptional regulators^14^.

In summary, our work provides a comprehensive analysis at single-cell resolution of the involvement of perivascular adipose tissue in diet-induced hypertension, including extensive alterations in cell type specific gene expression, cell type composition, cell-cell interactions, and transcriptional networks regulating these transitions. This work and associated data also serves as a rich resource for further study of this complex and fascinating tissue, and its role in vascular health and dysfunction.

## METHODS

### Animal Models and Tissue Collection

Dahl SS male and female rats were purchased from Charles River Laboratory at 3 weeks of age. Animals were placed on control diet (Research Diets, #D12450J) or high fat diet (Research Diets, #D12492) upon arrival and housed in a 12:12 light dark light cycle for 8 or 24 weeks, with food and water available *ad libitum*. Use and care of animals complied with National Institutes of Health Guide for the Care and Use of Laboratory Animals (2011) and was approved by the MSU Institutional Animal Care and Use Committee (PROTO202000009). The study design and reporting follow the Animal Research: Reporting of In Vivo Experiments (ARRIVE) guidelines^44^.

Due to the vital nature of adipose depots used in these snRNA-seq experiments, separate cohorts were utilized for the different time point tissue collections. Weights and MAPs for both the cohorts and specific rats used in the snRNA-seq experiments are therefore reported for transparency.

#### Blood Pressure measurements:

Tail cuff plethysmography was used to measure blood pressure at the 8 and 24 week time point (CODA Non-Invasive blood pressure system, Kent Scientific, Torrington CA USA). Both systolic and diastolic pressures were measured, allowing for calculation of mean arterial pressure (MAP). At the same time, weights of the rats were recorded. We report BP and weights of the rats from which the taPVAT was taken for experimentation (below) along with the rest of their cohort so that their context can be understood. Rats from these groups were randomly chosen for providing the tissue for snRNA-seq.

At the conclusion of the study (8 or 24 weeks on diet), rats were anesthetized with isoflurane and a bilateral pneumothorax was created prior to dissection of the thoracic aorta. Under a stereomicroscope, a Silastic^®^-coated dish was placed on ice and filled with physiological salt solution [PSS in mM: NaCl 130; KCl 4.7; KH_2_PO_4_ 1.18; MgSO_4_ • 7H_2_O 1.17; NaHCO_3_ 14.8; dextrose 5.5; CaNa_2_EDTA 0.03, CaCl_2_ 1.6 (pH 7.2)]. The taPVAT was removed from the vessel then snap frozen in liquid nitrogen and stored at −80° C.

### Nuclei Isolation and Flow Cytometry

Nuclei from frozen aortic perivascular adipose tissue were isolated with a modified protocol (dx.doi.org/10.17504/protocols.io.bkacksaw) of the Single Nuclei and Cytosol Isolation Kit for Adipose Tissues/Cultured Adipocytes (Invent Biotechnologies, #AN-029). Briefly, ~150 mg of taPVAT and BAT were minced on a glass plate with small scissors and transferred to a sterile 1.5 mL microcentrifuge tube. On ice, samples were homogenized in N/C Buffer (from kit) using the supplied pestle. Samples were transferred to filter cartridges in collection tubes (from kit) and incubated, with the caps open, at −20oC for 30 minutes. Samples were centrifuged, filters discarded and tubes closed, vortexed, then centrifuged again. Supernatant was removed and nuclei were resuspended in Wash and Resuspension buffer (1X PBS with 1.0% BSA, 0.2 U/μL RNAse inhibitor) containing DAPI (10 μg/mL). Samples were filtered using a 40-um strainer and immediately sorted using a BD FACSAria IIu (BD Biosciences, San Jose, CA) at the MSU Pharmacology and Toxicology Flow Cytometry Core (facs.iq.msu.edu/), targeting 50,000 events/sample. Sorted nuclei were immediately processed with the 10X Chromium Next GEM Single Cell 3’ Reagent Kit v 3.1 (Dual Index) according to manufacturer’s recommended protocol (CG000315, Rev D), using the maximum sample volume of 43.2 μL. The generated cDNA was quantified, and size distribution determined using a D5000 ScreenTape assay on an Agilent 4200 TapeStation. Twenty-five percent of the total cDNA was used to generate the single nuclei 3’ gene expression libraries. Libraries were amplified with individual 10X Sample Indexes from the Dual Index Plate TT Set A according to manufacturer’s protocol, using a SimpliAmp Thermal Cycler. Cleaned up samples were eluted into 35.0 μL Buffer EB, with the average fragment size of the libraries determined using a D1000 ScreenTape assay on an Agilent 4200 TapeStation.

### Library Quantification

The KAPA Library Quantification Kit was used to quantify library samples, with samples diluted 1:1000, 1:5000, 1:10000, and 1:20000 in DNA Dilution Buffer (10 mM Tris-HCl, pH 8.0 −8.5 + 0.05% Tween 20). Six μL KAPA SYBR FAST qPCR Master mix and 4 μL of 1) no template control (RNase-free water), 2) each diluted sample, or 3) kit supplied DNA Standard were pipetted into a standard 96-well PCR plate, in triplicate, and the plate covered with an adhesive film to prevent sample evaporation. The plate was centrifuged for 1 minute at 1000 rpm in an Eppendorf 5804 centrifuge and placed in a QuantStudio 6 Flex Real-Time PCR System. The following conditions were run: 1 cycle of 95°C 5 minutes; 35 cycles of 95°C 30 seconds, 60°C 45 seconds, followed by a standard melt curve (to ensure the lack of primer dimers). The KAPA Library Quantification Data Analysis Template was utilized to determine the concentration of the undiluted libraries, which were submitted to Novogene for 150bp paired-end sequencing at a target depth of 50,000 reads/nuclei on a NovaSeq6000.

### Computational Analysis of Single-cell Data

The quality of the raw reads was initially checked using FastQC v0.11.7. Then, sequencing files were aligned to the rat reference genome (Rnor 7.2) using the 10X Genomics Cell Ranger v.7.1.0 pipeline^45^. The resulting h5ad files of aligned reads were then preprocessed with the R packages SoupX^15^ and scDblFinder^16^ to remove ambient RNA and doublets respectively. The parameters used for initial quality control before running SoupX and scDblFinder were the same as outlined in the “Single Cell Best Practices” manual^46^ except for the cut off for the percentage of mitochondrial gene counts. We used a 3% cut-off instead of a 10% cut-off since we had lower mitochondrial counts due to our use of single-nuclei sequencing instead of single-cell. SnRNA-seq is expected to exclude the sources for mitochondrial RNA. Individual samples were further preprocessed using the scanpy package^47^ with cutoffs for the minimum number of genes per cell and minimum number of cells per gene. Genes found in 3 or fewer cells were removed, as were cells with fewer than 100 measured transcripts. Cells that had greater than 15000 reads or 4500 measured genes were also discarded. The package scDblFinder gives each cell a score between 0 and 1 based on how likely it thinks that cell is a doublet. Cells assigned a 1 are automatically labeled as doublets, to be more conservative about our approach, cells with a score of 0.5 or greater were removed. After these quality control steps, the anndata objects for each sample were combined, and additional preprocessing with scanpy was done. Cells that were outside of 5 median absolute deviations from the medians of the log1p_n_genes_by_counts and log1p_total_counts were labeled outliers and discarded.The high-dimensional data was then visualized using principal component analysis (PCA)^17^ and Uniform Approximation and Projection (UMAP)^18^. Batch integration was performed with scvi-tools^48^ to remove technical noise from the reduced-dimension plots. Then clusters were assigned using the Leiden algorithm. We had an informed understanding of the types of cells that we might find thanks to our previous work on building a cell atlas of rat taPVAT^49^. We labelled Leiden clusters with cell types based on the expression of marker genes that had been previously reported to be found in adipose tissue (Figure 1A – 1B). Cell type annotation was first done at low resolution (i.e. immune cells) to identify major cell types, then refined via subclustering to identify higher resolution cell types (i.e. macrophages). Differential gene expression (DEG) analysis was done with biological replicates using the DESeq2 package implemented in python^50,51^. Upset and volcano plots were generated using a custom Python script available in this project’s GitHub repository. ShinyGO v0.81^52^ was used to perform pathway enrichment analysis, specifically, overrepresentation analysis. This calculated pathways in the Gene Ontology biological process 2023 database^53^ that the DEGs were associated with. Significantly upregulated DEGs (*log2fold change≥1 and p≤0.05)* were input to ShinyGO separately from significantly downregulated DEGs (*log2fold change≤−1 and p≤0.05)*.

### Cell-cell Communication analysis

We used NicheNet to analyze cell-cell communication among the different cell types within PVAT, predicting how ligands from sender cells influence target gene expression in receiver cells. NicheNet integrates expression data with a model of signaling and gene regulatory networks, constructing a weighted network of ligand-receptor, intracellular signaling, and gene regulatory interactions. This approach employs a Personalized PageRank algorithm to compute ligand-target regulatory potential scores, prioritizing ligands based on their ability to predict differentially expressed genes in receiver cells. Potential signaling pathways are inferred with model parameters optimized using Bayesian methods. Our analysis focused on the top 700 highly expressed genes to ensure robust identification of ligand-receptor-target gene interactions.

We used NICHES to map cell-cell interactions at a single cell level focusing on ligand receptor signaling within different groups. NICHES constructs an adjacency matrix to define potential cell connections and builds matrices to capture ligand receptor signaling, the effect of individual cells on each other, the overall system’s influence on a cell, and each cell’s impact on its surroundings. We then perform differential analysis to_identify unique pathways within cellular clusters and uncover fine grained signaling patterns.

We also applied the LIgand-receptor ANAlysis Framework (LIANA) to examine communication patterns. LIANA has functionalities to use tools like CellChat and CellPhoneDB to infer cell-cell communication by analyzing paired ligand-receptor expression across different cell types. CellChat builds on a ligand-receptor interaction database (CellChatDB) derived from KEGG^54,55^ and recent literature, identifying differentially expressed signaling genes and calculating intercellular communication probabilities using a mass action model and random walk propagation. CellPhoneDB uses compiled database of ligand-receptor interactions and integrates it with the expression data to find meaningful interactions between cells. Both the tools use p-values to determine statistically significant interactions.

### RNA velocity analysis

To obtain the RNA velocity for each gene in each cell and to create the velocity vector embedding figures, we used the standard scVelo workflow, with minor changes. We used velocyto^35^ to generate the unspliced and spliced RNA count matrices that scVelo requires to calculate RNA velocity. We provided velocyto with (1) a filtered list of barcodes that were obtained from CellRanger’s cell calling algorithm, (2) bam files for each rat that were also generated with CellRanger, and (3) the rat reference genome annotation file (gtf) obtained from Ensembl (Rnor 7.2).

Velocyto outputs a loom file that includes unspliced and spliced reads for each rat. The biological replicates from each of the eight experimental conditions were then merged using loompy^56^, a standard package recommended by velocyto’s workflow, resulting in eight loom files. Depending on the analysis, sex- and diet-specific loom files were merged with the main single nuclei RNA sequencing dataset, using scVelo’s built in functions. The dataset, now containing unspliced and spliced reads, was filtered to include only brown adipocytes with at least 100 genes and for genes that were present in at least three cells. The dataset was then processed and analyzed with scVelo’s dynamical modeling workflow to generate the RNA velocity plots and heatmaps.

Diffusion maps were constructed using Scanpy methods (i.e., sc.tl.diffmap) and RNA velocity vectors were plotted on the diffusion map embeddings.

### Gene Regulatory Network Analysis

To obtain gene regulatory networks (GRN) and perform simulated knockouts, we used the standard CellOracle^14^ workflow (link here). The dataset was preprocessed according to CellOracle’s standard workflow and brown adipocytes were selected. Since no scATACseq data was available, we used CellOracle’s built-in rat promoter base gene regulatory network, which provides baseline information for gene regulatory networks (GRNs) in rats. Using this built-in base promoter GRN, CellOracle builds a GRN model specific to our dataset based on user-defined clusters and calculates various network scores for transcription factors. For each analysis, GRNs were constructed for each condition (“time diet” cluster). For example, for each sex, 8-week control diet and 8-week high-fat diet groups would each have a unique GRN.

To identify transcription factors of possible importance, we used information from three graph centrality measures: (1) betweenness centrality, (2) eigenvector centrality, and (3) degree-out centrality. In each analysis (females or males, 8- or 24-weeks) the ratio of graph measures in the high-fat diet versus control diet was calculated for each transcription factor. Larger ratios mean that the specific graph centrality measure became more prominent in the high-fat diet group compared to the control diet group for that specific transcription factor. Graph centrality ratios are then sorted from largest to smallest and ranked by position, starting from zero. Larger ratios for each centrality measure, therefore, correspond to smaller rank values. Finally, the rank values for the three centrality measures are summed for each transcription factor. This summation is sorted from smallest to largest, and the three transcription factors with the smallest total value are chosen to be computationally knocked out. The combination of betweenness centrality, eigenvector centrality, and degree-out centrality suggests these transcription factors may be highly influential within the gene regulatory network and in enabling the transition from control diet associated gene expression states to HF diet gene expression states.

## Extended Data

**Extended Data Table 1.** Top common interactions between adipocytes and endothelial cells in 8-week male rats, specific to control and high-fat diet groups, identified across all cell-cell communication tools.

Control Group	High-Fat Group
Tgm2 – Adgrg1	Col4a2 – Itgb3
Nrg4 – Erbb4	Angptl4 – Itgb3
Flrt1 – Unc5b	Nid1 – Itgb3
Flrt1 – Unc5c	Thbs1 – Cd47
Nrxn1 – Dag1	Thbs1 – Lrp5
Alcam – Alcam	Thbs1 – Itgb1
Alcam – Nrp1	Thbs1 – Itgb3
C4b – Cd46	Thbs1 – Itga6
Efna5 – Epha3	Gnas – Tshr
Efna5 – Ephb1	Cdh2 – Cdon
Adam10 – Epha3	App – Flt1
Adam10 – Notch4	Angptl4 – Cdh11
Adam10 – App	Spon1 – App
Adam10 – Tspan14	Nrg2 – Nrp2
Adam10 – Tspan5	Ptprm – Ptprm
Ctsd – App	Itgb1 – Vcam1
Lrig1 – Erbb4	Gpc6 – Ptch1
Lrig1 – Met	Glg1 – UncSb
Nrg4 – Erbb4	Pros1 – F8
Timp3 – Kdr	Plxna2 – Sema6a

**Extended Data Table 2.** Top transcription factors identified by CellOracle for Females (8W top, 24W bottom)

Gene	Eigenvector Centrality	Degree Centrality Out	Betweenness Centrality	Total Score
**Npas2**	0	0	12	12
**Nr4a1**	4	4	5	13
**Foxo3**	2	17	0	19
**Ppargc1a**	6	3	10	19
**Smad3**	15	1	4	20
**Nfia**	1	7	15	23
**Hlx**	19	2	3	24
**Mecom**	3	5	17	25
**Ppara**	9	9	8	26
**Hivep1**	5	10	14	29
**Ebf1**	13	6	11	30
**Fosl2**	8	16	7	31
**Cebpb**	11	11	9	31
**Nfib**	16	14	2	32
**Irf4**	18	15	1	34
**Bcl6**	12	8	16	36
**Nr4a2**	14	19	6	39
**Pbx1**	10	13	18	41
**Pparg**	17	12	13	42
**Thra**	7	18	19	44

Gene	Eigenvector Centrality	Degree Centrality Out	Betweenness Centrality	Total Score
**Nr4a1**	0	1	2	3
**Epas1**	3	2	0	5
**Nr4a2**	1	4	1	6
**Thra**	6	5	6	17
**Mecom**	2	3	14	19
**Irf4**	10	9	4	23
**Nfib**	4	17	3	24
**Meox2**	9	0	19	28
**Cebpb**	5	8	17	30
**Rxra**	7	7	16	30
**Nr1h2**	19	6	5	30
**Ppara**	8	14	10	32
**Srebf1**	13	11	8	32
**Hivep2**	14	12	9	35
**Ppargc1a**	16	10	11	37
**Bhlhe40**	17	13	7	37
**Ebf1**	15	15	12	42
**Pparg**	11	19	13	43
**Mafg**	12	18	18	48
**Rxrg**	18	16	15	49

**Extended Data Table 3.** Top transcription factors identified by CellOracle for Males (8W top, 24W bottom)

Gene	Eigenvector Centrality	Degree Centrality Out	Betweenness Centrality	Total Score
**Hlx**	3	1	4	8
**Bhlhe40**	2	4	3	9
**Ezh2**	0	0	12	12
**Ybx2**	6	2	10	18
**Hivep1**	5	13	5	23
**Tead1**	13	5	6	24
**Ppargc1a**	8	10	7	25
**Ppara**	4	11	13	28
**Ar**	10	17	2	29
**Hivep2**	9	3	17	29
**Tcf7l1**	1	14	14	29
**Stat5a**	12	18	1	31
**Ebf1**	15	8	8	31
**Trim28**	11	6	18	35
**Cebpb**	14	12	9	35
**Nr2c2**	16	9	11	36
**Mecom**	7	15	16	38
**Nfia**	19	19	0	38
**Nr4a1**	17	7	15	39
**Nr4a2**	18	16	19	53

Gene	Eigenvector Centrality	Degree Centrality Out	Betweenness Centrality	Total Score
**Cebpb**	3	0	2	5
**Nr4a3**	4	2	6	12
**Nr4a2**	2	1	11	14
**Hivep2**	0	7	8	15
**Irf4**	6	4	7	17
**Mecom**	15	3	5	23
**Bach2**	9	14	1	24
**Epas1**	10	11	4	25
**Hivep1**	1	16	9	26
**Ppard**	8	19	0	27
**Atf3**	5	5	19	29
**Npas2**	11	10	10	31
**Ebf1**	7	12	13	32
**Ppargc1a**	13	17	3	33
**Mitf**	12	6	17	35
**Nr4a1**	16	8	16	40
**Ppara**	19	9	12	40
**Nfia**	17	13	14	44
**Tcf7l1**	14	15	18	47
**Rxrg**	18	18	15	51

**Extended Data Figure 8:**
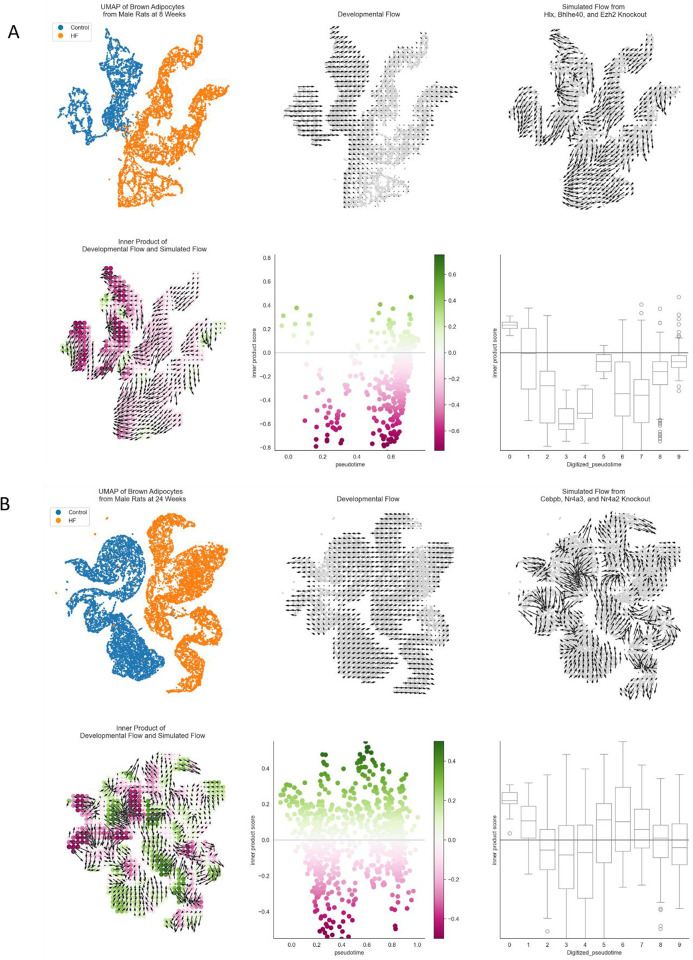
CellOracle transcription factor perturbation analyses for 8-week males (top) and 24-week males (bottom).

## Figures and Tables

**Table 1:** Top 10 cell-cell communication pathways across sex and diet.

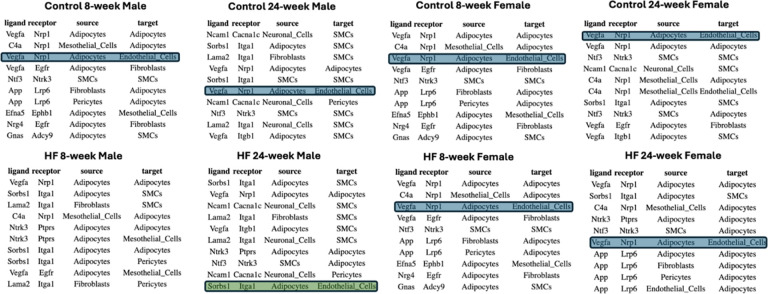

## Data Availability

Processed data can be conveniently queried through our implementation of the CellxGene Annotate web application located here: https://pvatcellatlas.azurewebsites.net/. Raw sequencing files will be made available through GEO upon publication. The GitHub repository for this project containing all notebooks and code will also be made available upon publication.
